# Exposure to a Combination of *Fusarium* Mycotoxins Leads to Lipid Peroxidation and Influences Antioxidant Defenses, Fatty Acid Composition of Phospholipids, and Renal Histology in Laying Hens

**DOI:** 10.3390/toxins16050226

**Published:** 2024-05-13

**Authors:** Szabina Kulcsár, Janka Turbók, György Kövér, Krisztián Balogh, Erika Zándoki, Omeralfaroug Ali, András Szabó, Miklós Mézes

**Affiliations:** 1 Department of Feed Safety, Institute of Physiology and Nutrition, Hungarian University of Agriculture and Life Sciences, Gödöllő Campus, H-2100 Gödöllő, Hungary; balogh.krisztian.milan@uni-mate.hu; 2 HUN-REN-MATE Mycotoxins in the Food Chain Research Group, Hungarian University of Agriculture and Life Sciences, H-7400 Kaposvár, Hungary; baloghne.zandoki.erika@uni-mate.hu (E.Z.); szabo.andras@uni-mate.hu (A.S.); 3 Agribiotechnology and Precision Breeding for Food Security National Laboratory, Institute of Physiology and Nutrition, Department of Physiology and Animal Health, Hungarian University of Agriculture and Life Sciences, H-7400 Kaposvár, Hungary; janka.turbok@gmail.com (J.T.); vomer2011@gmail.com (O.A.); 4 Department of Animal Science, Institute of Animal Breeding Sciences, Hungarian University of Agricultural and Life Sciences, H-7400 Kaposvár, Hungary; kover.gyorgy@uni-mate.hu

**Keywords:** oxidative stress, T-2 toxin, fumonisin B_1_, deoxynivalenol, lipid peroxidation, glutathione redox system, lipid composition, laying hens

## Abstract

The effects of combined short-term (3 days) exposure to *Fusarium* mycotoxins at both the EU recommended limit (T-2/HT-2 toxin: 0.25 mg/kg; DON/3-AcDON/15-AcDON: 5 mg/kg; FB_1_: 20 mg/kg) and twice the dose (T-2/HT-2 toxin: 0.5 mg/kg, DON/3-AcDON/15-AcDON: 10 mg/kg, and FB_1_: 40 mg/kg feed) on the kidneys of laying hens were examined. Our study aimed to investigate how these mycotoxins interacted with membrane lipid fatty acid (FA) composition and lipid peroxidation processes. It was observed that the levels of conjugated dienes and trienes were higher than the control in the low-mix group on day 3, and malondialdehyde concentration was higher on days 2 and 3. The proportion of phospholipid (PL) FAs showed that saturated and monounsaturated FAs increased. Still, both n3 and n6 polyunsaturated FAs decreased significantly on day 2 of exposure in the high-mix group. Among the n3 FAs, the level of docosahexaenoic (C22:6 n3) and among n6 FAs, arachidonic (C20:4 n6) acids decreased mainly on day 2 in the high-mix group. The results suggest that the combined exposure to *Fusarium* mycotoxins induced lipid peroxidation in the kidneys of laying hens, which resulted in marked changes in the PL FA profile. Histological examination revealed time- and dose-dependent increases as consequences of mycotoxin exposure.

## 1. Introduction

Mycotoxin contamination of feeds and foods poses risks to the health of both animals and humans, leading to financial setbacks within the food and feed industries and agricultural sector. In Europe, most cereals utilized in poultry feed contain *Fusarium* mycotoxins, including deoxynivalenol (DON), T-2/HT-2 toxin, and fumonisin B_1_ (FB_1_), with frequent instances of co-occurrence [1].

Poultry species are moderately susceptible to toxins produced by *Fusarium* moulds; while there may be no observable clinical symptoms of mycotoxin exposure, poultry may experience compromised production traits. The equal effects of trichothecenes, such as T-2 toxin and DON in laying hens, are feed refusal [2], reduced immune function [3], and diminished egg quality and production [4]. T-2 toxin specifically affects the kidneys among its target organs because it induces alterations in internal metabolism and disrupts multiple metabolic pathways, affecting energy utilization, amino acid metabolism, nucleotide synthesis, and oxidative stress responses [5]. This could be attributed to the toxin’s ability to interfere with key enzymatic processes and cellular functions. Additionally, the T-2 toxin may activate signaling pathways involved in stress responses and inflammation, cell apoptosis, and even fibrosis [6]. DON also causes reactive oxygen species (ROS)-mediated kidney damage in poultry [7].

FB_1_ can cause tissue damage and oxidative stress in the kidneys of chickens [8], reduced feed efficiency [9], and altered immune response [10]. The severity of the effects of mycotoxin exposure depends on their co-occurrence, contamination level, and exposure duration.

Because of their extensive distribution and harmful properties, the trichothecene mycotoxins, DON, its toxic metabolites (3-AcDON and 15-AcDON), and T-2/HT-2 toxin are severe contaminants [11]. Both trichothecenes contain an epoxide ring; therefore, they have high chemical reactivity [12]. Trichothecenes inhibit the protein synthesis of eukaryotic cells by inhibiting ribosomal functions [13]. Additionally, they prompt DNA damage, lipid peroxidation and oxidation of proteins (amino acids), disrupt the structural integrity of membranes, activate redox signaling pathways, and modify levels of antioxidants [14]. Inhibition of protein synthesis and lipid peroxidation causes mitochondrial dysfunction; therefore, oxygen free radicals, such as superoxide anion, are released from mitochondria, which can induce free radical formation and, consequently, oxidative stress [15].

FB_1_ is the most abundant form of fumonisin in naturally contaminated feed or food commodities [16]. The chemical structure of fumonisins is like that of sphingolipids and acts on sphingolipid metabolism as a sphingosine analog. Sphingolipids are essential in maintaining membrane integrity, cell growth, and differentiation [17]. Studies also showed that FB_1_ increased lipid peroxidation and ROS formation [18,19,20]. This process involves the oxidative degradation of lipids, particularly unsaturated fatty acids (FAs).

*Fusarium* mycotoxins, including T-2 toxin, DON, and FB_1_, exhibit notable individual and cumulative effects on the FA composition of liver and kidney membrane lipids in rats, pigs, and rabbits [21,22,23]. FA composition refers to the type and proportion of FAs in lipid molecules, such as phospholipids (PLs), triglycerides, and cholesterol esters [24]. Changes in the composition can be due to various factors, including mycotoxin exposure and its ROS-generating effect [21]. Oxidative stress from changes in antioxidant parameters and redox-sensitive gene expression have been identified as markers of oxidative stress [25,26,27]. It was hypothesized that the observed changes in antioxidant activity and gene expression may affect FA metabolism, potentially indicating a dynamic interaction between oxidative stress responses and lipid metabolic pathways. The purpose of the present study was to investigate the interaction between DON, T-2/HT-2 toxin, and FB_1_ on lipid peroxidation parameters, the FA composition of PLs, and histopathological alterations in the kidneys of laying hens.

## 2. Results

### 2.1. Body and Kidney Weight

The body weight (BW) remained consistent across all groups throughout the study period. Mycotoxin dose-dependent kidney relative weight difference was recorded on day 1, the control being significantly lower than the high-mix group. On the same day, there was a significant linear relationship between mycotoxin dose and relative kidney weight (R^2^ = 0.54, *p* < 0.001) (Table 1).

### 2.2. Indicators of Oxidative Imbalance and Antioxidant Function in the Kidney

**Figure 1:**
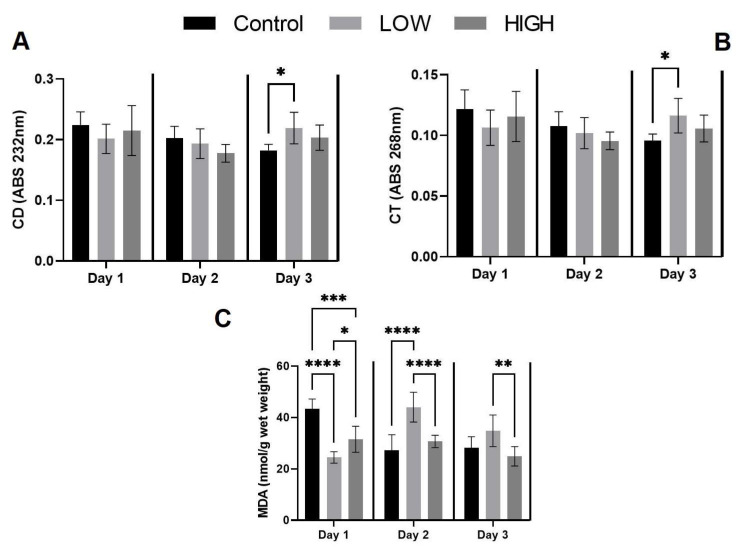
The oxidative (“(**A**)” CD, “(**B**)” CT, “(**C**)” MDA) parameters in the kidneys of laying hens (mean ± S.D.; *n* = 6). * *p*  <  0.05; ** *p*  <  0.01; *** *p*  <  0.001; **** *p*  <  0.0001. Low-mix group: T-2/HT-2 toxin: 0.25 mg/kg; DON/3-AcDON/15-AcDON: 5 mg/kg; FB_1_: 20 mg/kg, High-mix group: T-2/HT-2 toxin: 0.5 mg/kg, DON/3-AcDON/15-AcDON: 10 mg; FB_1_:40 mg/kg feed.

The conjugated diene (CD) and conjugated triene (CT) levels were significantly elevated by the third day in the low-mix group, in comparison to the control (Figure 1). The concentration of malondialdehyde (MDA), measured as thiobarbituric acid reactive substances (TBARS), reflecting the final stage of lipid peroxidation, exhibited a notable decrease in response to low mix exposure on day 1, in contrast to both the control and high-mix groups. After 48 h, it decreased significantly in comparison to the control and high-mix groups. This significant increase remained at the end of the experiment (Figure 1). No notable alterations were observed in the indicators of the glutathione system (reduced glutathione [GSH], glutathione peroxidase [GPx]) among the experimental groups (Table A1).

### 2.3. The Composition of Fatty Acids within Phospholipids of the Kidney

#### 2.3.1. Saturated Fatty Acyl Chains

A time-dependent increase in exposure was found for C14:0 (myristic acid). On day 2, the highest mycotoxin dose increased the C14:0 proportion in the kidney PLs, but this was not confirmed on day 3 (Table A2). The proportion of palmitic acid (C16:0) increased markedly in parallel with aging, for all groups. Mycotoxin dose dependence was statistically significant on days 2 and 3, but the changes were not systematic. Stearic acid (C18:0) provided a time-dependent decrease in all experimental groups, but in the intoxicated ones, this turned back to an increase. Mycotoxin dose altered stearic acid proportion significantly, but changes on days 2 and 3 were contradictory. Exposure time-associated decreases were found for arachidic acid (C20:0) in the control and low-mix groups. However, mycotoxin dose-dependent changes on the consecutive study days were not systematic; they were statistically significant. The proportion of behenic acid (C22:0) showed slight time-dependent decreases (control and low-mix groups), but again, proportional alteration on days 2 and 3 was contradictory. The presence and alteration of lignoceric acid (C24:0) in the renal PLs were negligible. The proportion of total saturated FAs increased with exposure time, but increased proportions by mycotoxin dose were only detected at day 2 (high-mix vs. control and low-mix groups).

#### 2.3.2. Monounsaturated Fatty Acids

The relative amount of palmitoleic acid (C16:1 n7) increased with exposure time in all experimental groups. Still, only day 2 resulted in a dose-dependent change, with the high-mix group showing a higher C16:1 n7 proportion on this day (Table A3). Fully identical alterations were found in oleic acid (C18:1 n9). The proportion of vaccenic acid (C18:1 n7) increased in all experimental groups with exposure time. Mycotoxin dose-associated proportional differences were not systematic. In this study, for all other monounsaturated FAs, gondoic (C20:1 n9), erucic (C22:1 n9), and nervonic (C24:1 n9) acids, there were no alterations recorded. Total monounsaturated FAs showed exposure-time-associated increases in all experimental groups, but the effect of mycotoxin dose was only detected on day 2, increasing the total monounsaturated FAs.

#### 2.3.3. Polyunsaturated Fatty Acyl Chains

The proportion of alpha-linolenic acid (C18:3 n3) showed exposure-time-associated fluctuation in the high-mix group and there was a rise corresponding to the dose on day 2 (Table A4). Eicosatrienoic acid (C20:3 n3) and eicosatetraenoic acid (C20:4 n3) were not responsive, regardless of exposure time or treatment. Eicosapentaenoic acid (C20:5 n3, EPA) decreased with the time of mycotoxin exposure, with the lowest values being reached on day 2. On the same day, the high mix had a significantly lower EPA proportion compared with the control. Meanwhile, *n*-3 docosapentaenoic acid (C22:5 n3) remained unchanged, and docosahexaenoic acid (C22:6 n3, DHA) decreased over exposure time in all groups. The toxin dose on day 2 resulted in a proportional modification; the high-mix group showed lower DHA levels in the kidney PLs in contrast to both the control and low-mix groups. A fully identical alteration pattern was found for the total n3 proportion.

Linoleic acid (C18:2 n6) proportions in the control and low mix-groups fluctuated over time, but mycotoxin treatment did not affect this acid (Table A4). In contrast, the proportion of gamma-linolenic acid (C18:3 n6) increased over exposure time in the control and low-mix groups. In contrast, mycotoxin dose-associated changes detected on days 2 and 3 were contradictory (increase vs. decrease) among the experimental groups. A mycotoxin dose-associated increase was found for eicosadienoic acid (C20:2 n6) but was only proven on day 2 in the high-mix group. Meanwhile, dihomo-gamma-linolenic acid (C20:3 n6) was unchanged in every regard, and the arachidonic acid (C20:4 n6) proportion reduced markedly over exposure time and showed a toxin dose-associated proportional drop (high mix vs. control and low mix). The proportion of docosatetraenoic acid (C22:4 n6, adrenic acid) decreased over time in all groups and showed a toxin dose-dependent decrease on day 2 (high mix vs. control and low mix). The proportion of n6 docosapentaenoic acid (C22:5 n6) showed a similar alteration to that detected for adrenic acid. The proportion of total n6 FAs decreased over time, and on day 2, the high mix caused a decrease in the proportion of total n6 FAs.

**Figure 2:**
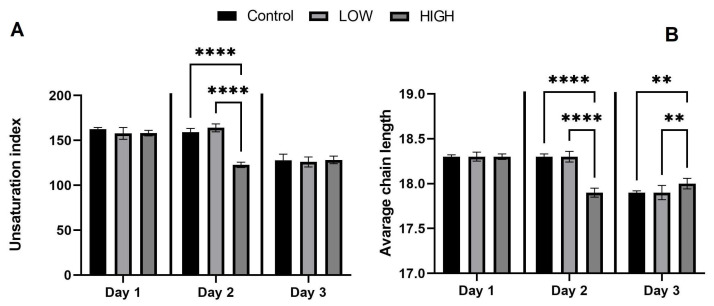
Changes in the unsaturation index dependent on exposure time and mycotoxin dose (**A**) and the mean length of fatty acid chains (**B**); ** *p*  <  0.01; **** *p*  <  0.0001. Low-mix group: T-2/HT-2 toxin: 0.25 mg/kg; DON/3-AcDON/15-AcDON: 5 mg/kg; FB_1_: 20 mg/kg feed, high-mix group: T-2/HT-2 toxin: 0.5 mg/kg, DON/3-AcDON/15-AcDON: 10 mg; FB_1_:40 mg/kg feed.

The unsaturation index (UI) declined dramatically over time, along with a reduction in the average chain length, which was most expressed in the high group (Figure 2).

### 2.4. Renal Tissue Histopathology

Table 2 presents the average total lesion scores for each experimental group and day. Across the experimental days, no differences were observed in mycotoxin dose association. Yet, within the high-mix group, a trend of increasing total lesion scores was noted as the trial progressed. In the histological sections, analyzed individually, altogether eight types of pathological symptoms were recognized: (S1) tubular cell detachment restricted to the perivascular area; (S2)multifocal tubular cell detachment; (S3) faint staining of the cytoplasm in tubular epithelial cells; (S4) multifocal discrete infiltration of mononuclear cells into the interstitium; (S5) focal discrete infiltration of mononuclear cells into the interstitium; (S6) swelling and degeneration of tubular epithelial cells; (S7) hydropic degeneration of tubular epithelial cells restricted to the perivascular area; (S8) karyopyknosis within tubular epithelial cells.

#### The Correlation between Histopathological Observations and Phospholipid Fatty Acid Profiles

Specific histopathological changes were identified, and the PL FA profile components (individual FAs and calculated FA indices) were examined for potential correlations. The results of this test are presented in Table 3 and show that Symptom 1 (tubular cell detachment restricted to the perivascular area) had significant positive correlations with saturated FAs (myristic and palmitic acids and total saturation) and a negative correlation with eicosapentaenoic acid, an n3-type FA. Interestingly, Symptom 2 (multifocal tubular cell detachment) correlated with an n6-type FA of minor contribution.

## 3. Discussion

Although the liver serves as the main site for metabolizing numerous mycotoxins, such as trichothecenes, it is worth noting that the kidneys also play a role in additional detoxification processes in poultry [28]. For instance, FB_1_ is recognized for its nephrotoxic impact, as it inhibits ceramide synthase, leading to an accumulation of sphinganine and the disturbance of cellular processes [29]. Furthermore, exposure to FB_1_ can trigger oxidative stress within renal cells, leading to ROS generation, subsequent oxidative damage to cellular constituents, and the release of pro-inflammatory cytokines [30]. T-2 toxin inhibits protein synthesis, disrupts mitochondrial function, and generates reactive oxygen species, leading to widespread metabolic disturbances and oxidative damage throughout the body [31]. DON exposure also leads to cell damage and impairs protein synthesis, causing gastrointestinal and kidney lesions in poultry [32]. Multi-mycotoxin exposure has a dose-dependent effect on the relative kidney weight, as observed on day 1, previously described in the case of the combination of aflatoxin and DON in laying hens [33]. The biochemical studies of the kidney indicated activated lipid peroxidation in the low-mix group on the second and third days of the experiment. This finding suggests that multi-mycotoxin exposure, in an individual dose at the European Union (EU)-recommended thresholds, increased oxidative stress in the kidneys, causing lipid peroxidation. Short-term exposure of laying hens to increased doses of T-2 toxin resulted in higher levels of MDA and GSH in the kidneys [26]. On the second day of the study, significant changes in FA proportions were detected concomitantly with the escalating multi-mycotoxin dosage.

There was a reduction in the ratio of unsaturated fatty acids and the mean chain length in the kidneys of the high-mix group, in contrast to both the control and low-mix groups, which was directly supported by the decrease in the ratio of arachidonic acid to adrenic acid (C20:4 n6/C22:4 n6, elongation) on day 2 parallel with the increasing toxin dose. Due to the high-dose mycotoxin combination, the antioxidant system was unable to completely neutralize oxygen free radicals. Consequently, lipid peroxidation was triggered, primarily impacting polyunsaturated FAs, which are prone to oxidative harm. As a result, their proportion decreased within total FAs. The results of the histopathology analysis also showed more lesions in the kidneys of high-dose-treated birds. Exposure to T-2 toxin induced pathological alterations in the kidneys of broiler chickens, such as vacuolar degeneration in the tubular epithelium, often accompanied by pyknotic nuclei [7]. Histopathological alterations were attributed to the influence of FB_1_ in Japanese quail after FB_1_ treatment [34]. The increased occurrence and severity of lesions are linked to mycotoxin exposure. These lesions could detrimentally impact the health of affected animals and lead to immunosuppression [35].

It is crucial to recognize that changes induced by mycotoxins in FA composition and the levels of indicators of lipid peroxidation might fluctuate based on various factors such as mycotoxin type, concentration, length of exposure, and the body’s antioxidant capacity. In addition, other factors, including the equilibrium between pro-oxidants and antioxidants, as well as the function of antioxidant enzymes, could affect the overall oxidative condition and the consequences of lipid peroxidation.

## 4. Conclusions

The changes in the lipid profile of laying hens’ kidneys after 3 days of mycotoxin exposure included alterations in the composition of PL FAs, changes in levels of lipid peroxidation markers such as MDA, variations in the ratio of saturated to unsaturated FAs, and potential disruptions in membrane integrity due to oxidative stress-induced damage. This study observed higher levels of CDs and CTs, as well as increased MDA concentration, at the European Union’s recommended threshold levels for individual mycotoxins. Additionally, the proportion of PL FAs increased in saturated and monounsaturated FAs, along with a decrease in both n3 and n6 polyunsaturated FAs. These results highlight the importance of monitoring mycotoxin exposure levels and their potential effects on oxidative stress and poultry kidney health, which will inform strategies for improved management practices and overall flock welfare.

## 5. Materials and Methods

### 5.1. The Experimental Setup and Its Parameters

In this study, 60 Tetra SL laying hens were used, each 49 weeks old, showing an average daily egg production rate of 90%. The hens were segregated into three treatment categories: control, low-mix, and high-mix, with each group comprising 18 animals. Additionally, six animals were designated as the absolute control group at the start of the experiment. Three treatment groups (control, low, and high) were formed, with 18 animals in each group, and an additional 6 animals served as the absolute control on day 0 of the experiment. The laying hens were fed and had access to drinking water ad libitum during the investigation. The composition of the poultry diet included 89.20% dry matter, 16.10% crude protein, 5.50% crude fiber, 2.50% ether extract, 4.12% calcium, 0.79% lysine, 0.38% methionine, 0.48% available phosphorus, 0.17% sodium, 0.71% methionine + cysteine, and 11.97 MJ/kg metabolizable energy. The animals were accommodated on deep litter under a natural light cycle of 12 h light and 12 h dark. After 12 h of feed deprivation, a 3-day feeding trial began. The laying hens were exposed to differing intensities of multi-mycotoxin contamination in their feed, including both low and high concentrations. In the low mix, there were 0.25 mg of T-2/HT-2 toxin, 5 mg of DON/3-AcDON/15-AcDON, and 20 mg/kg of FB_1_, whereas the high mix contained 0.5 mg of T-2/HT-2 toxin, 10 mg of DON/3-AcDON/15-AcDON, and 40 mg/kg feed of FB_1_. The experiment utilized the term “low mix” to denote mycotoxin levels that fell within the proposed limits set by the EU [36]. This dosage aimed to simulate mycotoxin contamination encountered in practical scenarios, such as through feed ingredients potentially affected by environmental variables or inadequate storage conditions [1]. Conversely, the “high-mix” dosage was intentionally formulated to induce observable alterations within a 3-day experimental framework. Kidneys play a role in the excretion of mycotoxins; post-mortem kidneys were collected on days 1, 2, and 3 of the experiment. Samples were taken randomly from 6 animals form each of the groups and stored at −70 °C until they were prepared for analysis.

### 5.2. Mycotoxin Assessment and Production

The feed underwent intentional contamination with T-2 toxin, FB_1_ and, DON. T-2 toxin originated from *Fusarium sporotrichioides* (NRRL 3299), FB_1_ from *Fusarium verticillioides* (MRC 826), and DON from *Fusarium graminearum* (NRRL 5883) cultivated on maize grain substrate. Culturing fungi on corn substrate was the same as natural contamination. The quantification of mycotoxin levels in the experimental feeds were quantified with the Shimadzu 2020 LCMS system, as described previously [37]. High-resolution chromatographic separation was achieved using an XB-C18 Kinetex analytical column (100 × 2.1 mm, 2.6 µm; Phenomenex, Torrance, CA, USA) at a 0.3 mL/min. flow rate. The column was maintained at a temperature of 40 °C throughout the analysis. Gradient elution employed eluent A, containing 0.1% formic acid and 0.005 M ammonium formate, and eluent B, containing 0.1% formic acid in acetonitrile. The gradient program began with eluent B at 10% and gradually increased linearly over 8 min. until it reached 100%. After that, the column underwent a 3 min wash with pure eluent B, followed by a linear reversion to the initial conditions over 1 min, and finally, the column was re-equilibrated for 3 min with 10% eluent B.

The examined mycotoxin levels in the experimental feed were below the limit of quantification (LOQ), and the concentration in the control feed was also below the LOQ (Table 4).

### 5.3. Methods of the Measurement of the Markers of Glutathione System and Lipid Peroxidation

CD and CT levels were assessed via analyzing the absorption spectra at wavelengths of 232 nm (CD) and 268 nm (CT), after the isolation of lipid content from the samples using 2,2,4-trimethylpentane (Sigma, St. Louis, MO, USA) [38]. MDA levels were determined using the 2-thiobarbituric acid method [39]. The TBARS were expressed as MDA based on the calibration curve of 1,1,3,3 tetraethoxy propane (Fluka, Buchs, Germany) [40].

The GSH concentration of the supernatant fraction (10,000 g) of kidney homogenates was assessed using the Sedlak and Lindsay method [41].

GPx activity was evaluated in the supernatant fraction (10,000 g) of kidney homogenates using an endpoint direct assay [42].

The quantification of GSH levels and GPx activity was standardized to the protein concentration of the supernatant fraction using Folin–Ciocalteu phenol reagent [43].

### 5.4. Analysis of Lipids

Kidney samples underwent homogenization in a solution consisting of chloroform and methanol in a ratio of 2:1 (*v*/*v*) at a 20-fold sample volume, as described by Folch et al. [44]. High-purity solvents (Merck-Sigma-Aldrich, Schnelldorf, Germany) were used, and 0.01% *w/v* butylated hydroxytoluene was included to inhibit FA oxidation. Ten milligrams of total extracted lipids were transferred to glass chromatographic columns containing 300 mg silica gel (230–400 mesh) for fractionation, using the Leray et al. method [45]. Neutral lipids were extracted with 10 mL of chloroform, then washed with 15 mL acetone–methanol (9:1 *v*/*v*), while total PLs were eluted with 10 mL pure methanol. The PL fraction underwent evaporation under a stream of nitrogen and was methylated using Christie’s base-catalysed NaOCH_3_ method [46].

FA methyl esters were quantified using the method described previously [37]. The results are presented as the weight percentage of total FA methyl esters.

### 5.5. Preparation of Histological Samples

Renal samples were fixed in 10% neutrally buffered formalin followed by embedding in paraffin. Sections of 5 μm were then obtained using a microtome and stained with hematoxylin–eosin for examination under a light microscope.

### 5.6. Histopathological Evaluation

The primary pathological changes were assessed and graded on their degree and extent as follows: 0 = absence of alteration, 1 = minimal/small scale/few, 2 = moderate degree/medium scale/moderate number, 3 = marked/extensive/numerous.

### 5.7. Data Analysis

Data are reported as mean ± standard deviation (S.D.). Group mean data were subjected to analysis of variance (ANOVA) followed by Tukey’s post hoc test to identify differences between groups. The variable used for grouping was the dose of mycotoxin (df = 2). Linear regression analysis was employed to assess the dose–response relationship. In the statistical analyses, significance was determined when *p*-values were less than 0.05. Histological lesions were statistically analyzed using two-way ANOVA, with exposure time (day) and mycotoxin dose as the two factors. The correlation between continuous PL FA and the ranked histological data was assessed using Spearman’s rho rank correlation method. All analyses were performed using IBM SPSS 29.0 (2022) software.

### 5.8. Ethical Allowance

The research followed the guidelines set out in the European Communities Council Directive (86/609 EEC), which establishes standards aimed at safeguarding the well-being and ethical treatment of animals utilized for scientific research purposes.

## Figures and Tables

**Table 1:** Somatic data of the laying hens during the intoxication study (mean ± S.D.; *n* = 6).

	Exp. Day	Control	Low Mix	High Mix	*p*	Observed Power
Bodyweight (g)	0	1645.8 ± 317.6	1645.8 ± 317.6	1645.8 ± 317.6		
	1	1590.8 ± 209.5	1537.5 ± 138.9	1709.2 ± 158.1		
	2	1699.5 ± 73.4	1597.0 ± 120.3	1615.8 ± 123.7		
	3	1652.8 ± 172.6	1626.2 ± 77.2	1677.3 ± 105.2		
Relative kidney weight (% of BW)	0	0.31 ± 0.07 ^a^	0.31 ± 0.07 ^a^	0.31 ± 0.07 ^a^		
	1	0.36 ± 0.08 ^aA^	0.48 ± 0.10 ^abAB^	0.52 ± 0.06 ^bB^	0.012	0.807
	2	0.61 ± 0.09 ^b^	0.52 ± 0.10 ^b^	0.57 ± 0.14 ^b^	0.13	0.181
	3	0.65 ± 0.08 ^b^	0.62 ± 0.21 ^b^	0.57 ± 0.16 ^b^	0.696	0.099
*p*		0.001	0.29	0.746		
Observed power		1	0.245	0.089		

^a,b^: small superscript letters denote differences within each group across successive experimental days (*p* < 0.05) ^A,B^: capital superscript letters indicate differences between the control, low-mix, and high-mix groups on specific experimental days *(p* < 0.05); low-mix group: T-2/HT-2 toxin: 0.25 mg/kg; DON/3-AcDON/15-AcDON: 5 mg/kg; FB_1_: 20 mg/kg, high-mix group: T-2/HT-2 toxin: 0.5 mg/kg, DON/3-AcDON/15-AcDON: 10 mg; FB_1_:40 mg/kg feed.

**Table 2:** The mean total lesion scores (S1-S8) across the experimental days and mycotoxin doses (mean ± SD).

	Control	Low Mix	High Mix
*Kidney*			
Day 1 Day 2 Day 3	3.67 ± 3.14 1.50 ± 1.38 2.50 ± 1.98	4.00 ± 2.83 1.83 ± 1.33 3.83 ± 2.64	1.83 ± 1.33 ^a^ 3.83 ± 1.72 ^ab^ 4.50 ± 1.87 ^b^

^a,b^: distinct superscript letters within a row indicate a statistically significant distinction. (*p* < 0.05); low-mix group: T-2/HT-2 toxin: 0.25 mg/kg; DON/3-AcDON/15-AcDON: 5 mg/kg; FB_1_: 20 mg/kg feed; high-mix group: T-2/HT-2 toxin: 0.5 mg/kg, DON/3-AcDON/15-AcDON: 10 mg; FB_1_:40 mg/kg feed.

**Table 3:** The Spearman correlations between individual FAs and the histological indicators of renal pathology.

Symptom/FA		C14:0	C16:0	C20:2 n6	C20:5 n3	SFA
**S1**	Coefficient of correlation	0.404	0.41		−0.4	0.407
	*Sig. (two-tailed)*	0.02	0.02		0.02	0.02
**S2**	Coefficient of correlation			0.521		
	*Sig. (two-tailed)*			0.05		

**Table 4:** The concentration of mycotoxins in the feed (mg/kg).

Group	T-2/HT-2	DON/3-AcDON/15-AcDON	FB_1_
Control Low mix High mix	<0.01 0.13/- 0.29/-	<0.01 0.67/0.56/0.01 2.70/0.89/0.05	<0.01 20.00 40.30

**Figure A1:**
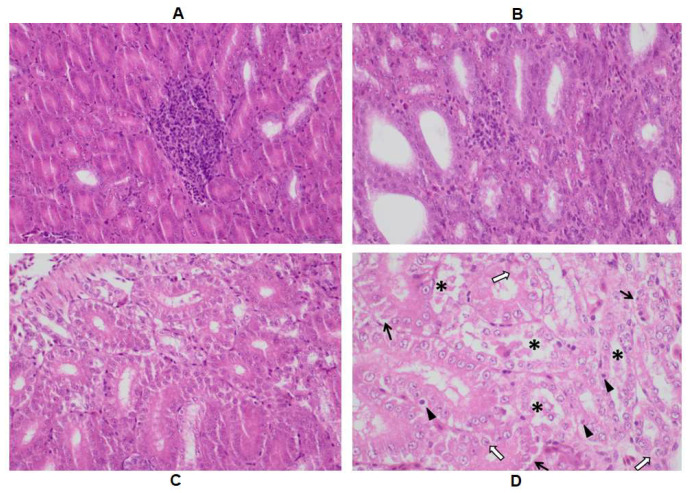
Results of histological examination: (**A**) marked focal mononuclear cell infiltration in the interstitium (H-E; 400X); (**B**) mild mononuclear cell infiltration in the interstitium surrounded with normal tubuli (H-E; 400X); (**C**) multifocal tubular damage with marked cellular detachment (H-E; 400X); (**D**) marked tubular lesions in the kidney with cellular detachment (black arrow), hydropic degeneration (white arrow), and karyopyknosis (arrowhead). Detached cells and cellular debris can be seen in multiple tubuli (asterisk)—H-E; 600X.

## Data Availability

The raw data supporting the conclusions of this manuscript will be made available by the authors, without undue reservation, to any qualified researcher.

## Appendix A

**Table A1:** The glutathione redox system parameters in the kidneys of laying hens (mean ± S.D.; *n* = 6).

Reduced Glutathione (μmol/g Protein)				
	Day 0	Day 1	Day 2	Day 3
Control	12.92 ± 1.18	13.05 ± 1.25	14.76 ± 1.14	10.60 ± 1.24
Low mix		13.62 ± 0.65	10.21 ± 2.59	13.54 ± 1.04
High mix		15.07 ± 1.34	11.56 ± 2.12	11.62 ± 0.96
**Glutathione Peroxidase** (**U/g Protein**)				
	**Day 0**	**Day 1**	**Day 2**	**Day 3**
Control	5.48 ± 0.94	6.92 ± 0.76	8.00 ± 0.06	9.33 ± 1.31
Low mix		7.06 ± 0.59	9.51 ± 2.95	13.15 ± 2.31
High mix		7.57 ± 0.33	10.16 ± 2.29	10.61 ± 1.54

**Table A2:** The composition of saturated fatty acids within renal phospholipids.

Fatty Acid	Exp. Day	Control	Low MIX	High Mix	Sig.	O. Power
**C14:0**	D_0	0.14 ± 0.02 ^a^	0.14 ± 0.02 ^a^	0.14 ± 0.02 ^a^		
	D_1	0.14 ± 0.02 ^a^	0.13 ± 0.03 ^a^	0.14 ± 0.01 ^a^	0.618	0.117
	D_2	0.14 ± 0.02 ^aA^	0.13 ± 0.02 ^aA^	0.21 ± 0.02 ^cB^	<0.001	1
	D_3	0.20 ± 0.02 ^b^	0.20 ± 0.02 ^b^	0.18 ± 0.02 ^a^	0.330	0.222
**Sig.**		<0.001	<0.001	<0.001		
**O. power**		1	0.994	1		
**C16:0**	D_0	18.6 ± 0.91 ^a^	18.6 ± 0.91 ^a^	18.6 ± 0.91 ^a^		
	D_1	18.8 ± 0.60 ^a^	18.9 ± 1.33 ^a^	19.0 ± 1.03 ^a^	0.945	0.057
	D_2	18.6 ± 0.45 ^aA^	17.7 ± 1.30 ^aA^	27.9 ± 1.29 ^cB^	<0.001	1
	D_3	26.9 ± 1.97 ^bB^	25.4 ± 1.75 ^bAB^	24.7 ± 1.55 ^bA^	0.108	0.437
**Sig.**		<0.001	<0.001	<0.001		
**O. power**		1	1	1		
**C18:0**	D_0	21.2 ± 1.12 ^b^	21.2 ± 1.12 ^b^	21.2 ± 1.12 ^c^		
	D_1	20.7 ± 0.60 ^b^	21.2 ± 1.18 ^b^	21.0 ± 0.89 ^c^	0.655	0.108
	D_2	21.2 ± 0.36 ^bB^	21.8 ± 1.21 ^bB^	15.8 ± 1.08 ^aA^	<0.001	1
	D_3	16.4 ± 1.57 ^aA^	17.9 ± 1.43 ^aAB^	18.8 ± 2.03 ^bB^	0.073	0.515
**Sig.**		<0.001	<0.001	<0.001		
**O. power**		1	0.998	1		
**C20:0**	D_0	0.17 ± 0.02 ^b^	0.17 ± 0.02 ^b^	0.17 ± 0.02 ^b^		
	D_1	0.17 ± 0.01 ^bAB^	0.17 ± 0.01 ^bA^	0.16 ± 0.01 ^bB^	0.067	0.529
	D_2	0.17 ± 0.01 ^bB^	0.17 ± 0.01 ^bB^	0.13 ± 0.01 ^aA^	<0.001	1
	D_3	0.14 ± 0.01 ^aA^	0.15 ± 0.01 ^aA^	0.20 ± 0.04 ^cB^	0.003	0.938
**Sig.**		<0.001	<0.001	0.002		
**O. power**		0.996	0.993	0.963		
**C22:0**	D_0	0.11 ± 0.03 ^b^	0.11 ± 0.03 ^b^	0.11 ± 0.03 ^b^		
	D_1	0.10 ± 0.00 ^bAb^	0.08 ± 0.01 ^aA^	0.09 ± 0.00 ^aAB^	0.01	0.829
	D_2	0.09 ± 0.01 ^a^	0.10 ± 0.01 ^ab^	0.09 ± 0.01 ^b^	0.178	0.338
	D_3	0.09 ± 0.01 ^a^	0.09 ± 0.01 ^a^	0.10 ± 0.03 ^ab^	0.457	0.165
**Sig.**		0.006	0.183	0.467		
**O. power**		0.875	0.334	0.161		
**C24:0**	D_0	0.01 ± 0.00 ^b^	0.01 ± 0.00 ^b^	0.01 ± 0.00		
	D_1	0.01 ± 0.00 ^a^	0.01 ± 0.00 ^a^	0.01 ± 0.00	0.137	0.389
	D_2	0.01 ± 0.00 ^a^	0.01 ± 0.00 ^ab^	0.01 ± 0.00	0.784	0.082
	D_3	0.01 ± 0.00 ^ab^	0.01 ± 0.00 ^b^	0.01 ± 0.00	0.863	0.069
**Sig.**		0.098	0.019	0.557		
**O. power**		0.457	0.747	0.133		
**total saturated**	D_0	40.2 ± 0.34 ^a^	40.2 ± 0.34 ^a^	40.2 ± 0.34 ^a^		
	D_1	40.0 ± 0.55 ^a^	40.5 ± 0.61 ^a^	40.4 ± 0.65 ^a^	0.346	0.213
	D_2	40.2 ± 0.18 ^aA^	39.9 ± 0.61 ^aA^	44.1 ± 0.86 ^aB^	<0.001	1
	D_3	43.8 ± 0.46 ^b^	43.7 ± 0.60 ^b^	44.0 ± 1.19 ^b^	0.844	0.072
**Sig,**		<0.001	<0.001	<0.001		
**O. power**		1	1	1		

^a,b,c^: lower case letters indicate differences within the group between consecutive experimental days (*p* < 0.05); ^A,B^: capital letters indicate differences between groups on each individual experimental day; Sig.: significance, indicating the *p*-value. O. power: observed power, representing the probability of detecting a true effect when it exists. Low-mix group: T-2/HT-2 toxin; 5 mg/kg DON/3-AcDON/15-AcDON; 20 mg/kg FB_1_; high-mix group: 0.5 mg/kg T-2/HT-2 toxin, 10 mg DON/3-AcDON/15-AcDON, 40 mg/kg feed FB_1._

**Table A3:** The composition of monounsaturated fatty acids within renal phospholipids.

Fatty Acid	Exp. Day	Control				Low Mix				High Mix				Sig.	O. Power
**C16:1n7**	D_0	0.64	±	0.27	a	0.64	±	0.27	a	0.64	±	0.27	a		
	D_1	0.72	±	0.14	ab	0.70	±	0.24	a	0.77	±	0.09	ab	0.804	0.079
	D_2	0.74	±	0.14	abA	0.65	±	0.10	aA	1.00	±	0.26	bB	0.012	0.81
	D_3	0.91	±	0.18	b	1.01	±	0.23	b	0.90	±	0.13	b	0.532	0.14
**Sig.**		0.083				0.015				0.105					
**O. power**		0.488				0.776				0.442					
**C18:1n9**	D_0	13.4	±	1.82	a	13.4	±	1.82	b	13.4	±	1.82	a		
	D_1	12.9	±	0.67	a	13.7	±	2.05	b	14.0	±	1.24	a	0.397	0.189
	D_2	14.1	±	1.49	aA	12.6	±	0.95	bA	17.3	±	2.55	bB	0.001	0.973
	D_3	17.4	±	3.03	b	18.0	±	1.82	a	18.0	±	1.33	b	0.853	0.07
**Sig,**		0.004				<0.001				0.003					
**O. power**		0.92				0.999				0.931					
**C18:1n7**	D_0	2.56	±	0.67	a	2.56	±	0.67	a	2.56	±	0.67	a		
	D_1	2.93	±	0.14	a	2.71	±	0.33	a	2.93	±	0.21	a	0.224	0.294
	D_2	2.97	±	0.14	aA	2.76	±	0.28	aA	4.00	±	0.54	bB	<0.001	1
	D_3	3.66	±	0.25	bAB	3.90	±	0.25	bB	3.47	±	0.18	bA	0.019	0.748
**Sig.**		<0.001				<0.001				<0.001					
**O. power**		1				1				0.993					
**C20:1n9**	D_0	0.31	±	0.06		0.31	±	0.06		0.31	±	0.06			
	D_1	0.29	±	0.02		0.28	±	0.06		0.29	±	0.02		0.776	0.083
	D_2	0.32	±	0.04		0.29	±	0.05		0.32	±	0.08		0.533	0.14
	D_3	0.34	±	0.09		0.34	±	0.06		0.33	±	0.05		0.975	0.053
**Sig.**		0.395				0.169				0.384					
**O. power**		0.19				0.349				0.195					
**C22:1n9**	D_0	0.07	±	0.01	b	0.07	±	0.01		0.07	±	0.01			
	D_1	0.06	±	0.00	ab	0.05	±	0.01		0.06	±	0.01		0.419	0.18
	D_2	0.06	±	0.01	a	0.06	±	0.01		0.06	±	0.01		0.868	0.068
	D_3	0.06	±	0.01	a	0.06	±	0.01		0.06	±	0.01		0.866	0.068
**Sig.**		0.479				0.59				0.852					
**O. power**		0.157				0.124				0.071					
**C24:1n9**	D_0	0.01	±	0.00		0.01	±	0.00		0.01	±	0.00			
	D_1	0.01	±	0.00		0.01	±	0.00		0.01	±	0.00		0.311	0.233
	D_2	0.01	±	0.00		0.01	±	0.00		0.01	±	0.00		0.303	0.237
	D_3	0.01	±	0.00		0.01	±	0.00		0.01	±	0.00		0.496	0.151
**Sig.**		0.608				0.411				0.84					
**O. power**		0.119				0.183				0.072					
**total monounsaturated**	D_0	17.0	±	2.59	a	17.0	±	2.59	a	17.0	±	2.59	a		
	D_1	16.9	±	0.85	a	17.5	±	2.53	a	18.0	±	1.35	a	0.53	0.141
	D_2	18.2	±	1.62	aA	16.3	±	1.15	aA	22.7	±	3.25	bB	<0.001	0.99
	D_3	22.4	±	3.22	b	23.3	±	2.10	b	22.8	±	1.26	b	0.787	0.486
**Sig.**		0.001				<0.001				0.002					
**O. power**		0.971				1				0.952					

a,b: lower case letters indicate differences within the group between consecutive experimental days (*p* < 0.05); A,B: capital letters indicate differences between groups on each individual experimental days; Sig.: ‘significance, indicating the *p*-value. O. power: observed power, representing the probability of detecting a true effect when it exists. Low-mix group: T-2/HT-2 toxin: 0.25 mg/kg; DON/3AcDON/15-AScDON: 5 mg/kg; FB_1_: 20 mg/kg feed; high-mix group: T-2/HT-2 toxin: 0.5 mg/kg, DON/3-AcDON/15-AcDON: 10 mg, FB_1_:40 mg/kg feed.

**Table A4:** Polyunsaturated fatty acid profile and calculated indices of the renal phospholipids.

Fatty Acid	Exp. Day	Control				Low Mix				High Mix				Sig.	O. Power
**C18:2n6**	D_0	11.3	±	2.19	a	11.3	±	2.19		11.3	±	2.19	a		
	D_1	12.1	±	0.46	ab	13.0	±	1.78		12.0	±	1.08	ab	0.292	0.244
	D_2	11.6	±	0.84	ab	12.7	±	1.98		14.0	±	3.17	b	0.196	0.32
	D_3	13.1	±	1.62	b	12.7	±	2.86		11.9	±	1.08	ab	0.565	0.13
**Sig.**		0.083				0.957				0.151					
**O. power**		0.489				0.055				0.371					
**C18:3n6**	D_0	0.07	±	0.03	a	0.07	±	0.03	a	0.07	±	0.03	a		
	D_1	0.08	±	0.01	a	0.08	±	0.02	a	0.08	±	0.01	a	0.785	0.082
	D_2	0.08	±	0.02	aA	0.07	±	0.01	aA	0.10	±	0.01	bB	0.006	0.881
	D_3	0.11	±	0.02	bB	0.11	±	0.02	bAB	0.09	±	0.01	abA	0.09	0.474
**Sig.**		0.005				0.006				0.002					
**O. power**		0.893				0.877				0.948					
**C18:3n3**	D_0	0.03	±	0.01		0.03	±	0.01		0.03	±	0.01	ab		
	D_1	0.03	±	0.00	B	0.02	±	0.00	A	0.02	±	0.01	aAB	0.082	0.491
	D_2	0.02	±	0.00	A	0.02	±	0.00	A	0.03	±	0.01	bB	0.007	0.867
	D_3	0.02	±	0.00		0.02	±	0.01		0.02	±	0.00	a	0.348	0.212
**Sig.**		0.076				0.41				0.062					
**O. power**		0.505				0.183				0.546					
**C20:2n6**	D_0	0.75	±	0.22		0.75	±	0.22		0.75	±	0.22			
	D_1	0.79	±	0.04		0.75	±	0.10		0.73	±	0.11		0.564	0.131
	D_2	0.73	±	0.05	A	0.81	±	0.06	AB	0.89	±	0.15	B	0.033	0.659
	D_3	0.86	±	0.17		0.82	±	0.09		0.71	±	0.12		0.167	0.351
**Sig.**		0.147				0.323				0.049					
**O. power**		0.376				0.226				0.589					
**C20:3n9**	D_0	0.40	±	0.21		0.40	±	0.21		0.40	±	0.21			
	D_1	0.37	±	0.09		0.31	±	0.23		0.43	±	0.14		0.453	0.166
	D_2	0.39	±	0.16		0.32	±	0.10		0.36	±	0.28		0.814	0.077
	D_3	0.30	±	0.16		0.36	±	0.21		0.39	±	0.14		0.699	0.098
**Sig.**		0.572				0.863				0.819					
**O. power**		0.129				0.069				0.076					
**C20:3n6**	D_0	4.40	±	1.46		4.40	±	1.46		4.40	±	1.46			
	D_1	4.44	±	0.83		3.94	±	0.62		4.12	±	0.45		0.421	0.179
	D_2	4.60	±	0.52		4.12	±	0.29		4.68	±	0.72		0.191	0.325
	D_3	4.43	±	0.58		4.41	±	0.38		4.62	±	0.35		0.672	0.104
**Sig.**		0.889				0.232				0.168					
**O. power**		0.065				0.288				0.35					
**C20:4n6**	D_0	21.3	±	2.29	b	21.3	±	2.29	b	21.3	±	2.29	b		
	D_1	21.2	±	0.69	b	19.7	±	2.30	b	19.9	±	0.64	b	0.175	0.341
	D_2	19.7	±	1.21	bB	21.5	±	1.44	bB	10.3	±	1.17	aA	<0.001	1
	D_3	12.0	±	2.47	a	11.3	±	1.63	a	12.3	±	0.99	a	0.656	0.108
**Sig.**		<0.001				<0.001				<0.001					
**O. power**		1				1				1					
**C20:3n3**	D_0	0.03	±	0.02		0.03	±	0.02		0.03	±	0.02			
	D_1	0.03	±	0.00		0.03	±	0.01		0.03	±	0.01		0.971	0.054
	D_2	0.03	±	0.01		0.03	±	0.01		0.04	±	0.02		0.536	0.139
	D_3	0.04	±	0.02		0.03	±	0.01		0.02	±	0.01		0.289	0.246
**Sig.**		0.361				0.859				0.341					
**O. power**		0.206				0.07				0.216					
**C20:4n3**	D_0	0.02	±	0.01		0.02	±	0.01		0.02	±	0.01	a		
	D_1	0.03	±	0.01		0.02	±	0.01		0.03	±	0.01	b	0.26	0.266
	D_2	0.03	±	0.01		0.03	±	0.00		0.03	±	0.01	ab	0.511	0.147
	D_3	0.03	±	0.01		0.03	±	0.01		0.03	±	0.01	b	0.775	0.083
**Sig.**		0.501				0.224				0.921					
**O. power**		0.15				0.294				0.06					
**C20:5n3**	D_0	0.27	±	0.05	b	0.27	±	0.05	c	0.27	±	0.05	c		
	D_1	0.23	±	0.04	ab	0.19	±	0.05	b	0.22	±	0.01	c	0.204	0.312
	D_2	0.22	±	0.03	aB	0.25	±	0.06	bAB	0.13	±	0.04	aA	0.001	0.972
	D_3	0.16	±	0.04	a	0.15	±	0.03	a	0.18	±	0.03	b	0.25	0.274
**Sig.**		0.014				0.006				0.001					
**O. power**		0.786				0.88				0.972					
**C22:4n6**	D_0	1.37	±	0.14	b	1.37	±	0.14	b	1.37	±	0.14	b		
	D_1	1.31	±	0.09	b	1.23	±	0.19	ab	1.35	±	0.09	b	0.32	0.228
	D_2	1.33	±	0.09	bB	1.31	±	0.22	abB	0.96	±	0.23	aA	0.007	0.866
	D_3	1.01	±	0.10	a	1.10	±	0.23	a	1.08	±	0.14	a	0.611	0.119
**Sig.**		<0.001				0.294				0.003					
**O. power**		1				0.243				0.939					
**C22:5n6**	D_0	1.31	±	0.36	b	1.31	±	0.36	b	1.31	±	0.36	b		
	D_1	1.14	±	0.28	b	1.23	±	0.34	ab	1.26	±	0.18	b	0.736	0.091
	D_2	1.27	±	0.36	bB	1.14	±	0.33	bAB	0.70	±	0.21	aA	0.014	0.789
	D_3	0.77	±	0.18	a	0.89	±	0.22	a	0.89	±	0.17	a	0.504	0.149
**Sig.**		0.02				0.162				<0.001					
**O. power**		0.735				0.357				0.992					
**C22:5n3**	D_0	0.22	±	0.03		0.22	±	0.03		0.22	±	0.03			
	D_1	0.26	±	0.02		0.24	±	0.05		0.23	±	0.03		0.541	0.138
	D_2	0.28	±	0.07		0.24	±	0.04		0.22	±	0.03		0.138	0.388
	D_3	0.25	±	0.07		0.25	±	0.04		0.22	±	0.02		0.413	0.182
**Sig.**		0.758				0.855				0.464					
**O. power**		0.087				0.07				0.162					
**C22:6n3**	D_0	1.25	±	0.25	b	1.25	±	0.25	b	1.25	±	0.25	b		
	D_1	1.14	±	0.20	b	1.22	±	0.20	b	1.17	±	0.15	b	0.747	0.089
	D_2	1.34	±	0.15	bB	1.25	±	0.15	bB	0.68	±	0.14	aA	<0.001	1
	D_3	0.80	±	0.12	a	0.76	±	0.08	a	0.82	±	0.15	a	0.663	0.106
**Sig.**		<0.001				<0.001				<0.001					
**O. power**		0.998				1				0.999					
**total n3**	D_0	1.82	±	0.26	b	1.82	±	0.26	b	1.82	±	0.26	b		
	D_1	1.71	±	0.22	b	1.72	±	0.23	b	1.71	±	0.17	b	0.987	0.052
	D_2	1.92	±	0.23	bB	1.81	±	0.14	bB	1.12	±	0.18	aA	<0.001	1
	D_3	1.31	±	0.20	a	1.23	±	0.13	a	1.29	±	0.18	a	0.735	0.091
**Sig.**		<0.001				<0.001				<0.001					
**O. power**		0.987				0.999				0.999					
**total n6**	D_0	40.5	±	2.63	b	40.5	±	2.63	b	40.5	±	2.63	b		
	D_1	41.1	±	0.48	b	40.0	±	2.53	b	39.4	±	1.40	b	0.271	0.258
	D_2	39.3	±	1.71	bB	41.7	±	1.50	bB	31.7	±	2.71	aA	<0.001	1
	D_3	32.3	±	2.76	a	31.4	±	2.03	a	31.6	±	1.02	a	0.739	0.09
**Sig.**		<0.001				<0.001				<0.001					
**O. power**		1				1				1					

a,b,c: lower case letters indicate differences within the group between consecutive experimental days (*p* < 0.05); A,B: capital letters indicate differences between groups on each individual experimental days; Sig.: significance, indicating the *p*-value. O. power: observed power, representing the probability of detecting a true effect when it exists. Low-mix” group: T-2/HT-2 toxin: 0.25 mg/kg; DON/3AcDON/15-AScDON: 5 mg/kg; FB_1_: 20 mg/kg feed; high-mix group: T-2/HT-2 toxin: 0.5 mg/kg, DON/3-AcDON/15-AcDON: 10 mg, FB_1_:40 mg/kg feed.
